# POLE/POLD1 mutation in non‐exonuclease domain matters for predicting efficacy of immune‐checkpoint‐inhibitor therapy

**DOI:** 10.1002/ctm2.524

**Published:** 2021-09-26

**Authors:** Yan‐Xing Chen, Zi‐Xian Wang, Shu‐Qiang Yuan, Teng‐Jia Jiang, You‐Sheng Huang, Rui‐Hua Xu, Feng Wang, Qi Zhao

**Affiliations:** ^1^ State Key Laboratory of Oncology in South China, Sun Yat‐sen University Cancer Center Collaborative Innovation Center for Cancer Medicine Guangzhou Guangdong 510060 China; ^2^ Precision Diagnosis and Treatment for Gastrointestinal Cancer Chinese Academy of Medical Sciences Guangzhou 510060 China

To the Editor:

With the development of immunotherapy, cancer treatment has stepped into a new era.^1^ Though POLE/POLD1 mutation (POL‐MUT) have been proven to be a promising marker in ICI treatment,^2^, ^3^, ^4^, ^5^ the predictive value of different types of POL‐MUTs has yet to be fully investigated. By classifying POL‐MUTs into exonuclease domain mutations (POL‐EDMs) and non‐exonuclease domain mutations (POL‐non‐EDMs) based on their locations and mutational types,^6^ we found that domain location is not a determining factor for the predictive value of POL‐MUTs, and POL‐non‐EDMs do matter in screening ICI‐sensitive tumor patients.

Through collecting and analyzing the mutation data of 43,890 patients from cBio‐Portal database, we found that most POL‐MUTs were evenly located on the POLE and POLD1 genes without an apparent tendency to gather in the exonuclease domain. (Figure S1A,B). The overall proportion of tumors with POL‐EDMs was 0.6%, while the number of those only with POL‐non‐EDMs was 2.4% (Figure 1A, Table S3). POL‐MUTs annotated as pathological variants (POL‐PVs) mainly occurred amongst EC and colorectal cancer (Figure S1C). Tumor mutation burden (TMB) of both POL‐EDM tumors and POL‐non‐EDM tumors was significantly higher than that of POL‐WT tumors (Figure 1B, Figure S2A, Tables S4), so was the fraction of ultra‐hypermutant (*p*‐value < .001) and hypermutant tumors (*p*‐value < .001). Some (*n* = 28) POL‐non‐EDMs occurred more than once and co‐occurred with hypermutant status (Figure S2B–D, Table S5 and S6). After removing tumors carrying POL‐PVs, mutational signatures associated with impairment of the POLE/POLD1 proofreading function^7^ were still detected in some POL‐MUT tumors, and some of them (*n = 48*) only carried POL‐non‐EDM (Figure 1C, Table S7). Overall, we speculated that a certain fraction of POL‐non‐EDMs is strongly correlated with higher TMB and corresponding mutational signatures.

**FIGURE 1:**
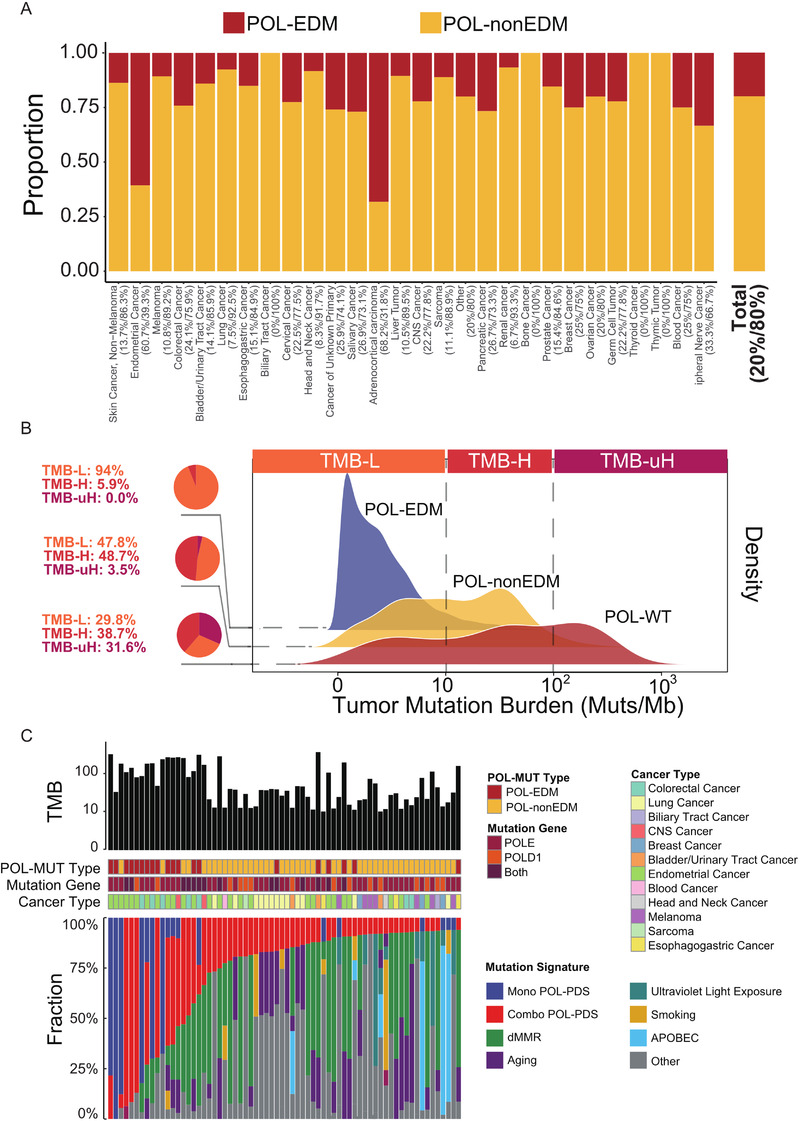
Prevalence and predicted functional impact of POL‐EDM and POL‐non‐EDM. (A) Proportion of POL‐EDM and POL‐non‐EDM tumors identified for the cohort and each cancer type. Numbers next to cancer types indicate the percentage of POL‐EDM tumors and POL‐non‐EDM tumors. “CNS cancer” means tumors in central nervous system. (B) TMB distribution in POL‐WT, POL‐non‐EDM, and POL‐EDM tumors in studies using WES (the dashed lines were used to denote the cutoff of TMB‐L (<10 Muts/Mb), TMB‐H (≥10 Muts/Mb), and TMB‐uH (≥100 Muts/Mb). Pie plot and the number on the left denote the proportion of TMB‐L, TMB‐H, and TMB‐uH tumors. (C) Mutational signatures in TMB‐H tumors carrying the POL‐PDS signature and POL‐MUTs without previous annotation as drivers. Each column described the features of one tumor sample, including its TMB, classification of POL‐MUT, mutation gene (“Both” means the sample carried *POLE* and *POLD1* mutations simultaneously), cancer type, and fraction of each detected mutational signature

Given that domain location did not sufficiently distinguish proofreading damaging mutations, we analyzed all nonsynonymous POLE/POLD1 mutations to explore its performance in predicting the clinical benefits of ICIs. With an integrated ICI treatment cohort of 1172 cancer patients (Table S1), we found that patients carrying POL‐MUTs achieved significantly higher objective response rate^8^ than POL‐WT patients (Figure 2A, 59.2% vs. 25.5%, *p*‐value < .001), even in both TMB‐high and TMB‐low subgroups (Figure 2B). Similarly, higher disease control rate (DCR) (74.1% vs. 52.4%, *p*‐value = .002; Figure 2C, Figure S3A), higher durable clinical benefit (DCB) rate (53.5% vs. 33.8%, *p*‐value = .013; Figure 2D, Figure S3B), longer progression‐free survival (PFS) (HR = 0.55, *p*‐adjust = .003; Figure 2E), and longer overall survival (OS) (HR = 0.62, *p*‐adjust = .03; Figure 2F) were all detected in POL‐MUT patients compared with POL‐WT patients. The predictive value was further validated by an independent cohort with OS (Figure S3C). Moreover, no survival difference was detected between POL‐MUT patients and POL‐WT patients in the non‐ICI treatment cohort (Figure S3D), suggesting that the OS benefits of ICIs were unlikely to be attributed to their general prognostic impacts.

**FIGURE 2:**
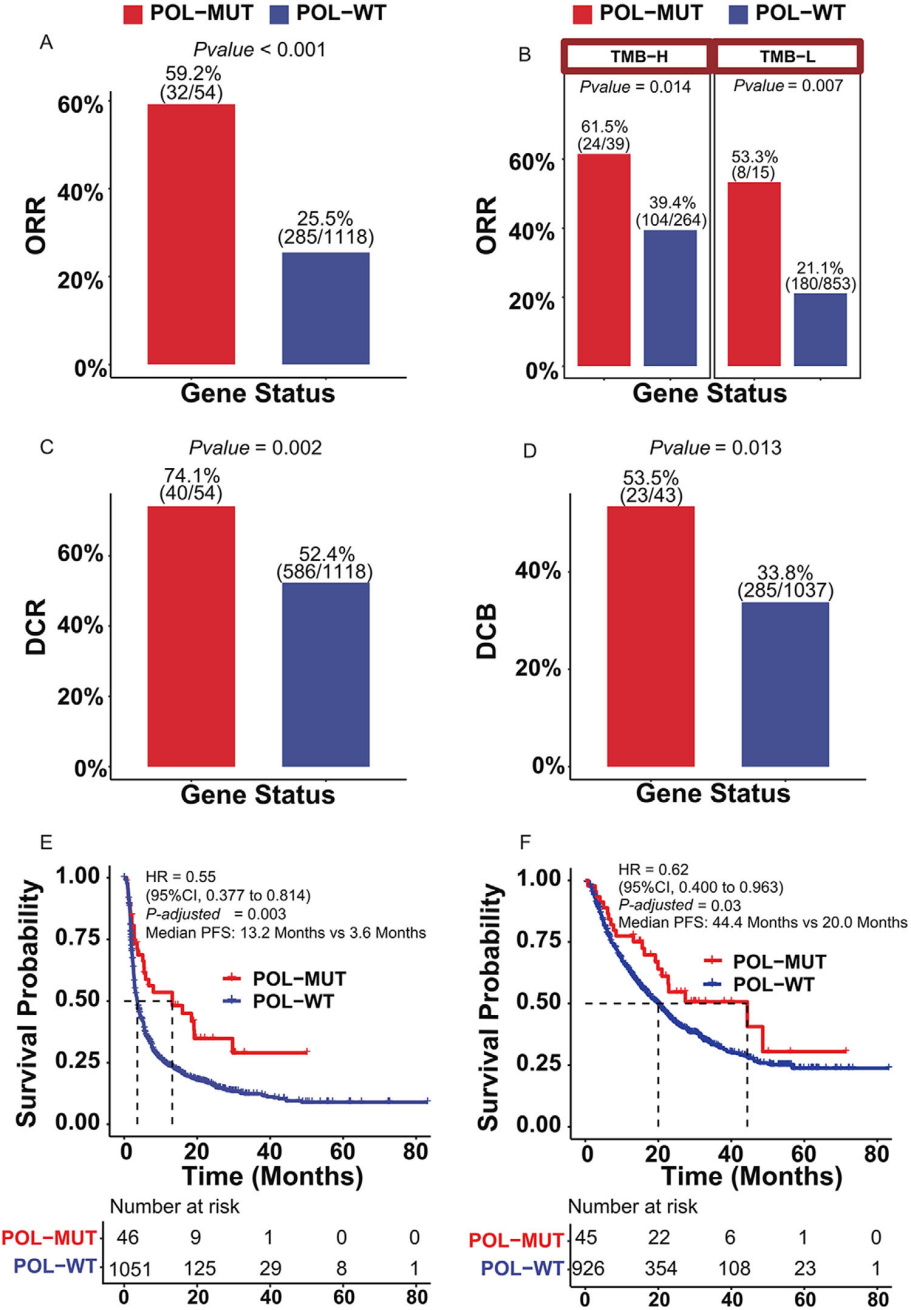
Association of unselected *POLE*/*POLD1* mutations and clinical outcomes in ICI treatment. (A) Comparison of objective response rate between POL‐MUT and POL‐WT patients after receiving ICI treatment. (B) Comparison of objective response rate between POL‐MUT and POL‐WT patients after receiving ICI treatment in TMB‐high and TMB‐low subgroup. (C) Comparison of disease control rate between POL‐MUT and POL‐WT patients after receiving ICI treatment. (D) Percentage of patients who derived DCB in the POL‐MUT and POL‐WT patients after receiving ICI treatment. (E) Comparison of progression‐free survival (PFS) between POL‐MUT patients and their WT counterparts in ICI treatment Cohort 1 by Kaplan–Meier estimates. (F) Comparison of overall survival (OS) between POL‐MUT patients and their WT counterparts in ICI treatment Cohort 1 by Kaplan–Meier estimates

Interestingly, POL‐MUTs in response patients did not gather on exonuclease domain (Figure 3A,B, Table S8), but evenly distributed on the whole proteins. Specifically, patients carrying POL‐non‐EDM achieved significantly higher ORR than POL‐WT patients (Figure 3C, 58.7% vs. 23.8%, *p*‐value < .001), even in TMB‐high and TMB‐low subgroups (Figure S4A). Similarly, compared with POL‐WT patients, significantly longer OS and PFS were also observed in POL‐non‐EDM patients (Figure S4B,C). The association between POL‐non‐EDM and benefit in ICI treatment was further validated by an independent cohort with OS (Figure 3D). Though patients carrying POL‐EDM also tended to derive more benefit than POL‐WT patients, the difference was not statistically significant, probably due to the limited POL‐MUT cases.

**FIGURE 3:**
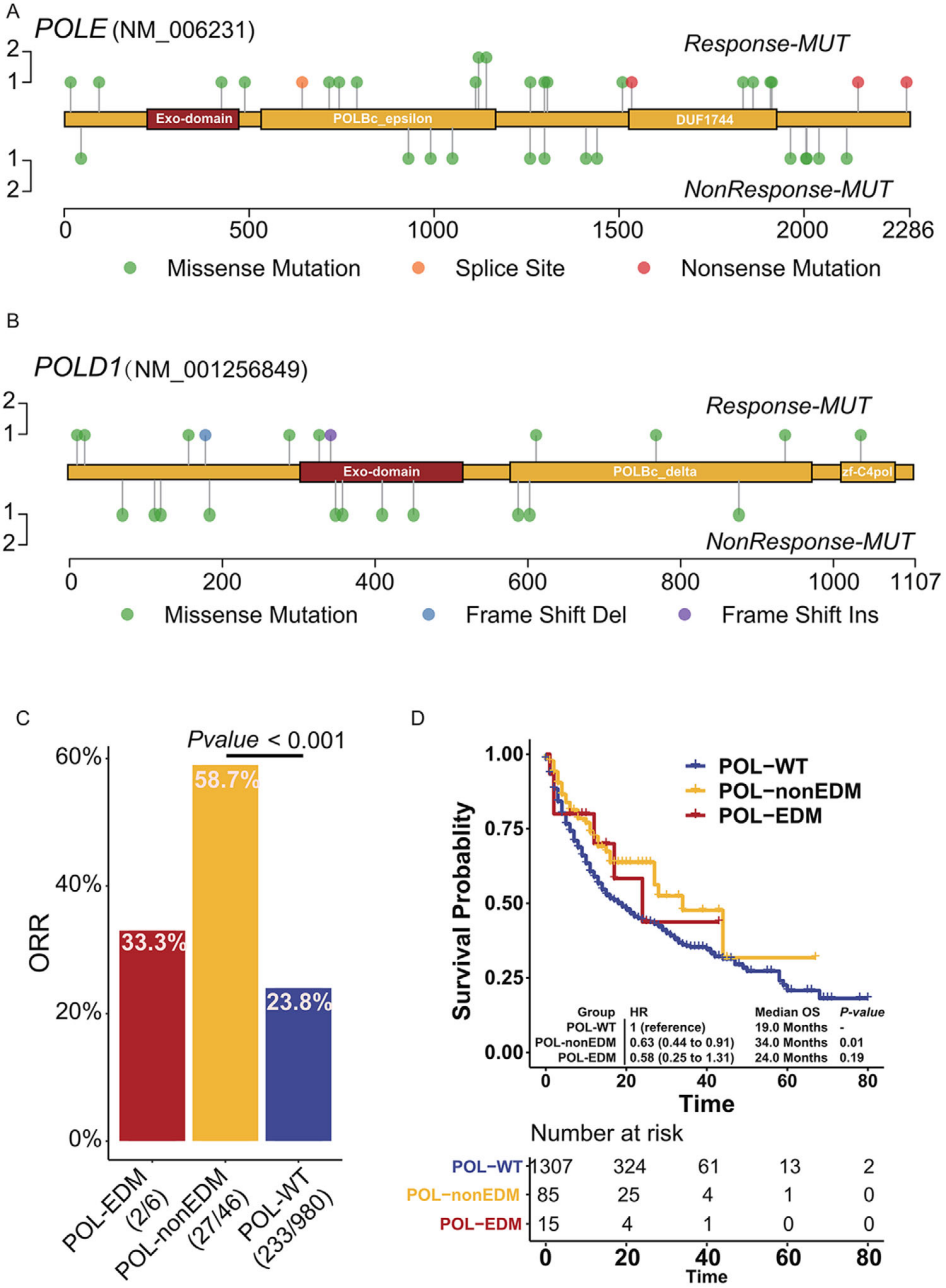
Association between different *POLE*/*POLD1* mutation types and clinical outcomes. (A) Positional distribution of *POLE* mutations in Cohort 1 with the responders annotated. (B) Positional distribution of *POLD1* mutations in Cohort 1 with the responders annotated. (C) Percentage of patients who derived objective response in the POL‐EDM, POL‐non‐EDM, and POL‐WT patients. (D) Comparison on overall survival (OS) among POL‐EDM, POL‐non‐EDM, and POL‐WT patients in ICI treatment Cohort 2 by Kaplan–Meier estimates

To further investigate the possible mechanism underlying the comparative predictive capability of POL‐non‐EDM and POL‐EDM, we analyzed the alteration of immune phenotypes in POL‐MUT tumors. As expected, the neoantigen burden in POL‐non‐EDM tumors was significantly higher than that in POL‐WT tumors (*p*‐value < .001), but significantly lower than that in POL‐EDM (*p*‐value < .001) tumors and MSI‐H tumors (*p*‐value < .001) (Figure 4A). In addition, POL‐MUT tumors were highly correlated with mutations involved in DNA damage repair (DDR) pathway, regardless of EDM or non‐EDM status (Figure 4B). But it is worth noting that large fraction of POL‐EDM tumors and POL‐non‐EDM tumors are MSS (Figure S5A). After removing MSI‐H tumors, both leukocyte infiltration and CD8+ T‐cell infiltration in POL‐non‐EDM tumors were higher than those in POL‐WT tumors (Figure 4C,D). For POL‐EDM tumors, leukocyte infiltration was comparable to that in POL‐WT tumors, whereas CD8+ T‐cell infiltration was significantly higher than that in POL‐WT tumors. Despite the higher neoantigen burden in POL‐EDM tumors, neither leukocyte infiltration nor CD8+ T‐cell infiltration was found to be different between POL‐EDM tumors and POL‐non‐EDM tumors (Figure 4C,D). After excluding hypermutant tumors, leukocyte infiltration and CD8+ T‐cell infiltration in POL‐non‐EDM tumors (but not POL‐EDM tumors, probably due to the limited sample size) remained higher than those in POL‐WT tumors (Figure 4E,F). Similar phenomenon was observed in immune‐associated pathway. (Figure 4G,H). Taken together, activated immunity in POL‐MUT tumors might not be fully attributed to a high‐TMB or high‐neoantigen burden.

**FIGURE 4:**
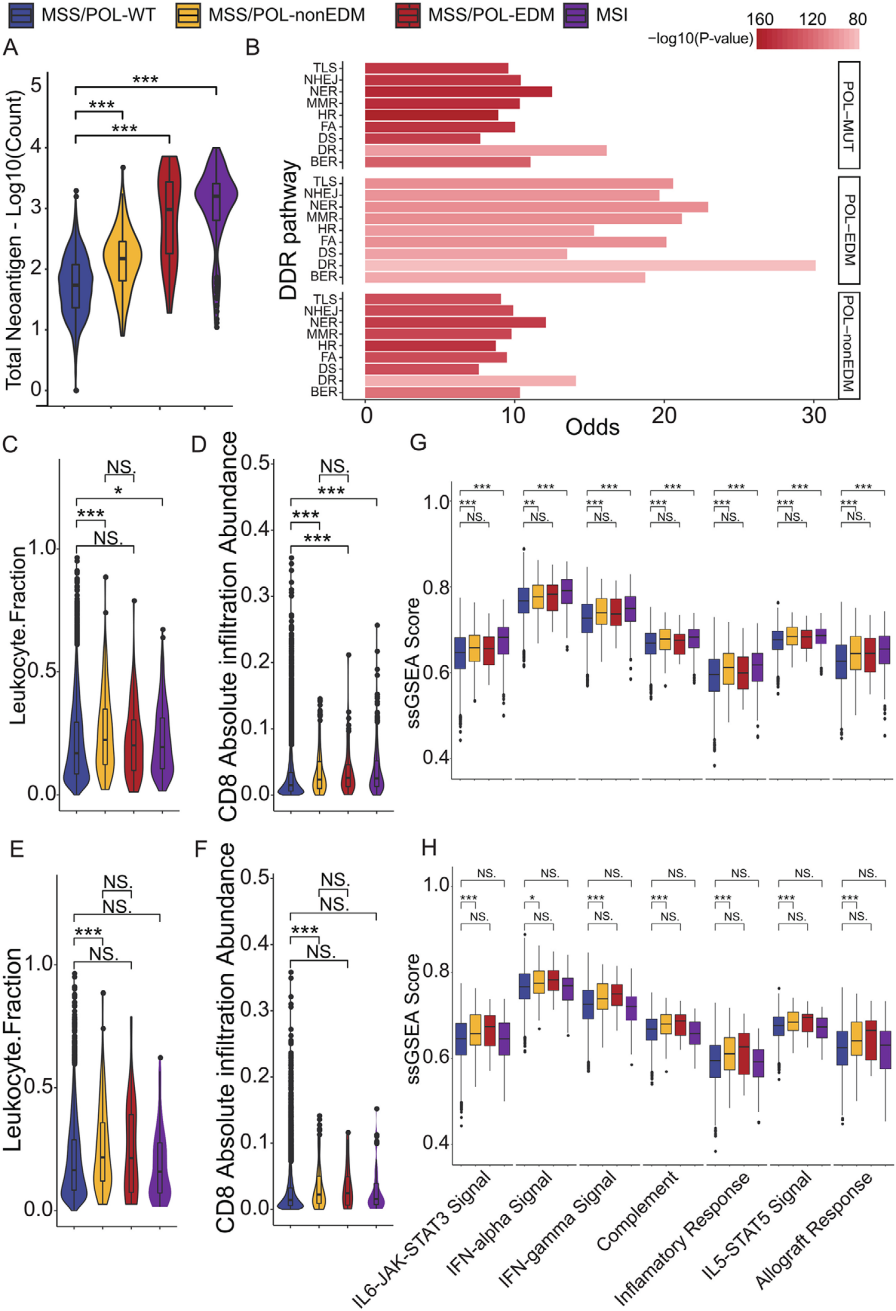
Immune phenotypes of POL‐MUT tumors. (A) Neoantigen burden in tumors with MSI‐H status, MSS/POL‐EDM, MSS/POL‐non‐EDM, and MSS/POL‐WT. (B) Correlation between DDR pathway mutations and POL‐MUT. Odds ratio (OR) greater than 0 indicates a positive correlation. Abbreviations: TLS, translesion synthesis; NHEJ, non‐homologous end joining; NER, nucleotide excision repair; MMR, mismatch repair; HDR, homology‐dependent recombination; FA, Fanconi anemia; DS, damage sensor; DR, direct repair; BER, base excision repair. (C) Leukocyte infiltration fraction in tumors with MSI‐H status (*n* = 402), MSS/POL‐EDM (*n* = 88), MSS/POL‐non‐EDM (*n* = 247), and MSS/POL‐WT (*n* = 9369). (D) CD8^+^ T‐cell infiltration in tumors with MSI‐H status, MSS/POL‐EDM, MSS/POL‐non‐EDM, and MSS/POL‐WT. (E) Leukocyte infiltration fraction in TMB‐L tumors with MSI‐H status (*n* = 83), MSS/POL‐EDM (*n* = 24), MSS/POL‐non‐EDM (*n* = 162), and MSS/POL‐WT (*n* = 8654). (F) CD8^+^ T‐cell infiltration in TMB‐L tumors with MSI‐H status, MSS/POL‐EDM, MSS/POL‐non‐EDM, and MSS/POL‐WT. (G) ssGSEA score of HALLMARK immune‐associated pathways in tumors with MSI‐H status, MSS/POL‐EDM, MSS/POL‐non‐EDM, and MSS/POL‐WT. (H) ssGSEA score of HALLMARK immune‐associated pathways in TMB‐L tumors with MSI‐H status, MSS/POL‐EDM, MSS/POL‐non‐EDM, and MSS/POL‐WT. The figure legend denting the color of (A) and (C–H) is on the top; *p‐value < .05; **p‐value < .01; ***p‐value < .001

Conclusively, with integrated cohorts containing 2862 cancer patients receiving immunotherapy, we found that both unselected POL‐MUTs and POL‐non‐EDMs could serve as biomarkers in screening responders in ICI treatments in addition to POL‐EDMs. Directly driving mutation accumulation^3^ and correlating with some internally or externally mutagenic factors are two known underlying mechanisms. Besides, POL‐non‐EDM is associated with an activated immune response, even in low‐TMB tumors, suggesting that some POL‐MUTs might be able to trigger an immune response via some patterns independent of hypermutant status, such as serving as neoantigens with high immunogenicity and directly connecting to immune pathways such as some DDR pathways. As most of the POL‐MUTs occurred out of the exonuclease domain, our findings expanded the understanding and use of POL‐MUTs in ICI treatment.

## CONFLICT OF INTEREST

The authors declare that they have no conflict of interest.

## Supporting information

Supplementary Table

Supplementary Material

Supplementary Figure
