# Supplementary material for: POLE/POLD1 mutation in non‐exonuclease domain matters for predicting efficacy of immune‐checkpoint‐inhibitor therapy
Source: Clin Transl Med. 2021 Sep 26;11(9):e524. doi: 10.1002/ctm2.524 (PMC8473642; doi:10.1002/ctm2.524)
Supplement: Supplementary file 2 — Supplementary Material [file CTM2-11-e524-s002.docx]

**Supplementary Materials**

**Methods:**

**Data collection and study design**

Information on *POLE* and *POLD1* mutations in the cBioportal cohort was directly downloaded from the cBioportal website [1] by searching all nonredundant cohorts on 2020-01-01. The sample ID, patient ID, mutation count of each sample, and other clinical features in all nonredundant cohorts were also downloaded from the search results on the website. By manually checking the matched sample ID and patient ID, and removing redundant samples, 45539 samples from 43890 patients were retained for further analysis. The variant information of all samples in the cBioportal were downloaded from its datahub [1], and redundant samples were removed by matching the cohort ID, patient ID, and sample ID obtained above. The sequencing method of each cohort was collected from the *meta_study.txt* of each study at cBioportal datahub [1].

To validate the predictive value of *POLE* and *POLD1* mutations in ICI treatment, cohorts with annotated response, PFS, OS and mutational data of patients receiving ICI treatment from 11 published studies [2-15] was collected and consolidated whose detailed information was shown in Supplementary Table 1. 10 cohorts that have efficacy annotation and survival information were consolidated as cohort1 while MSKCC cohort from Samstein et al. [15] that only have overall survival information was defined as cohort2 after removing redundant patients.

The clinical data and mutation information of the MSKCC non-ICI treatment cohort from the *msk_impact_2017* study [15] were downloaded from [cBioportal datahub](https://github.com/cBioPortal/datahub) [1]. TCGA RNA-seq data, mutation data, and clinical data were collected from the UCSC Xena data portal [16] (<https://xenabrowser.net)>. The MSI status of tumors in the TCGA cohort was collected from Russell et al.’s study [17].

**Grouping *POLE*/*POLD1* mutations**

According to the UniProt database, 269 to 471 amino acids of the POLE protein (ID: Q07864) and 304 to 517 amino acids of the POLD1 protein (ID: P28340) were from the regions of the exonuclease domain of two DNA polymerases [18]. Accordingly, missense mutations and in-frame insertions or deletions (INDELs) impacting the exonuclease domain of the POLE and POLD1 proteins were classified as POL-EDMs. Other mutations, including truncating mutations, missense mutations and in-frame INDELs not occurring in the exonuclease domain were classified as POL-nonEDMs. Tumors with POL-EDMs were defined as POL-EDM tumors, while tumors with POL-nonEDMs but without POL-EDMs were defined as POL-nonEDM tumors. Mutations already annotated in OncoKB [19] and a study by Campbell et al.’s [20] were defined as POL-pathogenic variant (POL-PV), while the others were defined as POL-nonpathogenic variant (POL-nonPV). Similarly, tumors with POL-PVs were defined as POL-PV tumors, while tumors with POL-nonPVs but without POL-PVs were defined as POL-nonPV tumors. When MSI-H was taken as a classification factor, tumors with MSI-H status were first classified as MSI-H tumors, and the rest were classified according to their POL-MUT status.

**Analysis of mutation-associated features**

We applied the *Lollipopplot* function in the R package ‘maftools’ [21] to present the regional location of each *POLE*/*POLD1* mutation. With ANNOVAR [22] and “dbnsfp30a” as references, SIFT [23] and Polyphen2-HDIV [24] were used to predict the functional impact of *POLE/POLD1* mutations on their encoding proteins. Only when a mutation was predicted as damaging in SIFT and damaging/probably damaging in Polyphen2-HDIV, it would be classified as a predicted damaging mutation.

**Mutation burden and mutation signature**

Only data sequenced by WES and MSK-IMPACT were included for the comparison of tumor mutation burden (TMB) and mutation signature. For samples from the cBioportal database, the mutation count of each sample was collected as mentioned above. For samples from the published ICI-treatment cohort, the mutation count of each sample was the sum of all nonsynonymous mutations. For samples sequenced by the MSK-IMPACT panel, TMB was calculated as the mutation count divided by the sum of the bases targeted by the panel (0.98, 1.06, and 1.22 Mb in the 341-, 410-, and 468-gene panels, respectively). For samples sequenced by WES, TMB was calculated as the mutation count divided by 38 Mb [25]. In addition, the TMB of TCGA samples was directly collected from Thorsson et al.’s study [26]. The mutation signatures were extracted from the mutation data by the R package ‘deconstructSigs’ [27], and only results in samples sequenced by WES with ≥10 Mut/Mb and samples sequenced by MSKCC-IMPACT with ≥100 Mut/Mb were used for the corresponding analysis. According to previous studies, the COSMIC mutation signatures SBS10 (CMSS10) [28] are specific mutation signatures directly associated with the impairment of *POLE*/*POLD1* proofreading function, while SBS14 (CMSS14) and SBS20 (CMSS20) [28, 29] are specific mutation signatures directly associated with the impairment of *POLE*/*POLD1* proofreading function combined with deficiency of mismatch repair (dMMR). Therefore, we considered CMSS10 as Mono*-POLE/POLD1* proofreading damagings signature (Mono POL-PDS) and combined14, and 20 as Combo-*POLE/POLD1* proofreading damaging signature (Combo POL-PDS),. CMSS1A and CMSS1B were combined as an aging signature [28]. CMSS7 was defined as the UA signature [28]. CMSS4 was defined as a smoking signature [28]. CMSS6, CMSS15, and CMSS21 were combined as a dMMR signature [28]. CMSS2 and CMSS13 were combined as an aging signature [28].

**Clinical outcomes**

The primary clinical outcomes were ORR, DCB, PFS, and OS. ORR was assessed using the Response Evaluation Criteria in Solid Tumors (RECIST) version 1.1. Clinical benefit was classified as a durable clinical benefit (DCB; complete response [CR]/partial response [PR] or stable disease [SD] that lasted >6 months) or no durable benefit (NDB, progressive disease [PD] or SD that lasted ≤6 months). PFS was assessed from the date the patient began immunotherapy to the date of progression or death from any cause. Patients who had not progressed were censored at the date of their last scan.

**Immune phenotype assessment**

Neoantigen burden was defined as the total predicted neoantigen count as determined by Thorsson et al [26]. CIBERSORT [30] was used to estimate the infiltration fraction of CD8+ T cells. The fraction of infiltrating leukocytes was obtained from Thorsson et al.’s study [26]. Gene sets of hallmark pathways were downloaded from <https://www.gsea-msigdb.org/gsea/index.jsp>. The R package ‘GSVA’ [18] was used to determine the single-sample gene set enrichment (ssGSEA) scores of each pathway in each sample. The gene list of DNA damage repair (DDR) pathways was downloaded from the following website: <https://www.mdanderson.org/documents/Labs/Wood-Laboratory/human-dna-repair-genes.html>. Mutations of a specific DDR pathway meant that at least one gene in the pathway was mutated. Only samples sequenced with WES were included to explore the correlation between DDR pathway mutations (excluding *POLE* and *POLD1*) and POL-MUT. Since a null value was not allowed in the least absolute shrinkage and selection operator (LASSO) regression analysis, and some of the samples in the ICI treatment cohort were sequenced with the MSK-IMPACT 341 gene panel (without *POLD1*), we only adopted mutations in DDR genes included in the panel as independent variables for the analysis.

**Statistical methods**

The chi-square test was used to investigate the predictive value of POL-MUT for ORR and DCB. Multivariable Cox proportional hazards models were used to determine the effect of POL-MUT status on survival outcomes (PFS and OS), which were adjusted for cancer types. Comparisons of numeric data were performed using the Wilcoxon rank-sum test. The correlation between DDR pathway mutations and POL-MUT was examined using Fisher’s exact test. P-values in multiple group comparisons were adjusted with the BH method. The nominal level of significance was set at 0.05 and all statistical tests were two-sided. Statistical analyses were performed using R v. 3.5.2 (http://www.r-project.org).

**References:**

1. Cerami E, Gao J, Dogrusoz U, Gross BE, Sumer SO, Aksoy BA et al. The cBio cancer genomics portal: an open platform for exploring multidimensional cancer genomics data. Cancer Discov 2012; 2: 401-404.

2. Janjigian YY, Sanchez-Vega F, Jonsson P, Chatila WK, Hechtman JF, Ku GY et al. Genetic Predictors of Response to Systemic Therapy in Esophagogastric Cancer. Cancer Discov 2018; 8: 49-58.

3. Rizvi H, Sanchez-Vega F, La K, Chatila W, Jonsson P, Halpenny D et al. Molecular Determinants of Response to Anti-Programmed Cell Death (PD)-1 and Anti-Programmed Death-Ligand 1 (PD-L1) Blockade in Patients With Non-Small-Cell Lung Cancer Profiled With Targeted Next-Generation Sequencing. J Clin Oncol 2018; 36: 633-641.

4. Rizvi NA, Hellmann MD, Snyder A, Kvistborg P, Makarov V, Havel JJ et al. Cancer immunology. Mutational landscape determines sensitivity to PD-1 blockade in non-small cell lung cancer. Science 2015; 348: 124-128.

5. Snyder A, Makarov V, Merghoub T, Yuan J, Zaretsky JM, Desrichard A et al. Genetic basis for clinical response to CTLA-4 blockade in melanoma. N Engl J Med 2014; 371: 2189-2199.

6. Van Allen EM, Miao D, Schilling B, Shukla SA, Blank C, Zimmer L et al. Genomic correlates of response to CTLA-4 blockade in metastatic melanoma. Science 2015; 350: 207-211.

7. Miao D, Margolis CA, Vokes NI, Liu D, Taylor-Weiner A, Wankowicz SM et al. Genomic correlates of response to immune checkpoint blockade in microsatellite-stable solid tumors. Nat Genet 2018; 50: 1271-1281.

8. Braun DA, Hou Y, Bakouny Z, Ficial M, Sant' Angelo M, Forman J et al. Interplay of somatic alterations and immune infiltration modulates response to PD-1 blockade in advanced clear cell renal cell carcinoma. Nat Med 2020; 26: 909-918.

9. Hellmann MD, Nathanson T, Rizvi H, Creelan BC, Sanchez-Vega F, Ahuja A et al. Genomic Features of Response to Combination Immunotherapy in Patients with Advanced Non-Small-Cell Lung Cancer. Cancer Cell 2018; 33: 843-852 e844.

10. Hugo W, Zaretsky JM, Sun L, Song C, Moreno BH, Hu-Lieskovan S et al. Genomic and Transcriptomic Features of Response to Anti-PD-1 Therapy in Metastatic Melanoma. Cell 2016; 165: 35-44.

11. Jung H, Kim HS, Kim JY, Sun JM, Ahn JS, Ahn MJ et al. DNA methylation loss promotes immune evasion of tumours with high mutation and copy number load. Nat Commun 2019; 10: 4278.

12. Kim ST, Cristescu R, Bass AJ, Kim KM, Odegaard JI, Kim K et al. Comprehensive molecular characterization of clinical responses to PD-1 inhibition in metastatic gastric cancer. Nat Med 2018; 24: 1449-1458.

13. Liu D, Schilling B, Liu D, Sucker A, Livingstone E, Jerby-Arnon L et al. Integrative molecular and clinical modeling of clinical outcomes to PD1 blockade in patients with metastatic melanoma. Nat Med 2019; 25: 1916-1927.

14. Wang F, Wei XL, Wang FH, Xu N, Shen L, Dai GH et al. Safety, efficacy and tumor mutational burden as a biomarker of overall survival benefit in chemo-refractory gastric cancer treated with toripalimab, a PD-1 antibody in phase Ib/II clinical trial NCT02915432. Ann Oncol 2019; 30: 1479-1486.

15. Samstein RM, Lee CH, Shoushtari AN, Hellmann MD, Shen R, Janjigian YY et al. Tumor mutational load predicts survival after immunotherapy across multiple cancer types. Nat Genet 2019; 51: 202-206.

16. Mary Goldman BC, Mim Hastie, Kristupas Repečka, Fran McDade, Akhil Kamath, Ayan Banerjee, Yunhai Luo, Dave Rogers, Angela N. Brooks, Jingchun Zhu, David Haussler. The UCSC Xena platform for public and private cancer genomics data visualization and interpretation. bioRxiv 2019.

17. Bonneville R, Krook MA, Kautto EA, Miya J, Wing MR, Chen HZ et al. Landscape of Microsatellite Instability Across 39 Cancer Types. JCO Precis Oncol 2017; 2017.

18. Hanzelmann S, Castelo R, Guinney J. GSVA: gene set variation analysis for microarray and RNA-seq data. BMC Bioinformatics 2013; 14: 7.

19. Chakravarty D, Gao J, Phillips SM, Kundra R, Zhang H, Wang J et al. OncoKB: A Precision Oncology Knowledge Base. JCO Precis Oncol 2017; 2017.

20. Campbell BB, Light N, Fabrizio D, Zatzman M, Fuligni F, de Borja R et al. Comprehensive Analysis of Hypermutation in Human Cancer. Cell 2017; 171: 1042-1056 e1010.

21. Mayakonda A, Lin DC, Assenov Y, Plass C, Koeffler HP. Maftools: efficient and comprehensive analysis of somatic variants in cancer. Genome Res 2018; 28: 1747-1756.

22. Wang K, Li M, Hakonarson H. ANNOVAR: functional annotation of genetic variants from high-throughput sequencing data. Nucleic Acids Res 2010; 38: e164.

23. Ng PC, Henikoff S. SIFT: Predicting amino acid changes that affect protein function. Nucleic Acids Res 2003; 31: 3812-3814.

24. Adzhubei IA, Schmidt S, Peshkin L, Ramensky VE, Gerasimova A, Bork P et al. A method and server for predicting damaging missense mutations. Nat Methods 2010; 7: 248-249.

25. Chalmers ZR, Connelly CF, Fabrizio D, Gay L, Ali SM, Ennis R et al. Analysis of 100,000 human cancer genomes reveals the landscape of tumor mutational burden. Genome Med 2017; 9: 34.

26. Thorsson V, Gibbs DL, Brown SD, Wolf D, Bortone DS, Ou Yang TH et al. The Immune Landscape of Cancer. Immunity 2018; 48: 812-830 e814.

27. Rosenthal R, McGranahan N, Herrero J, Taylor BS, Swanton C. DeconstructSigs: delineating mutational processes in single tumors distinguishes DNA repair deficiencies and patterns of carcinoma evolution. Genome Biol 2016; 17: 31.

28. Alexandrov LB, Kim J, Haradhvala NJ, Huang MN, Tian Ng AW, Wu Y et al. The repertoire of mutational signatures in human cancer. Nature 2020; 578: 94-101.

29. Haradhvala NJ, Kim J, Maruvka YE, Polak P, Rosebrock D, Livitz D et al. Distinct mutational signatures characterize concurrent loss of polymerase proofreading and mismatch repair. Nat Commun 2018; 9: 1746.

30. Newman AM, Liu CL, Green MR, Gentles AJ, Feng W, Xu Y et al. Robust enumeration of cell subsets from tissue expression profiles. Nat Methods 2015; 12: 453-457.

**Supplementary Figure Legends:**

**Supplementary Figure 1. Systematic profile of POL-MUT** (A) Location on the encoding protein and frequency of all *POLE* mutations collected from cBio-portal database. (B) Location on the encoding protein and frequency of all *POLD1* mutations collected from cBio-portal database. (C) Percentage of different cancer types among patients carried POL-MUTs previously annotated as pathological variants.

**Supplementary Figure 2. Association between POL-MUT and TMB.** (A) TMB distribution in POL-WT, POL-nonEDM and POL-EDM patients in studies using MSKCC-Impact Panels. (B) Lowest TMB detected in each POL-MUT. (Axis X is amino acid change of each POL-MUT and ranked by the amino acid position on POLE or POLD1; if a specific POL-MUT was only detected in one patient, the lowest TMB of the POL-MUT was the patient’s TMB; if a specific POL-MUT was detected in several tumors, the lowest TMB of the POL-MUT was the TMB of the patient who carried the lowest TMB; the size of the points on the lines depicts the frequency of each POL-MUT). (C) Lowest TMB detected in each *POLE* mutational location. (D) Lowest TMB detected in each *POLD1* mutational location.

**Supplementary Figure 3. Association of unselected *POLE*/*POLD1* mutations and clinical outcomes** **in ICI treatment.** (A) Proportions of patients achieved disease control in POL-MUT and POL-WT patients in TMB-high and TMB-low subgroup in ICI treatment. (B) Proportions of patients achieved durable clinical benefit in POL-MUT and POL-WT patients in TMB-high and TMB-low subgroup in ICI treatment. (C) Kaplan-Meier estimates of overall (OS) in Cohort2 comparing patients with POL-MUT with their respective WT counterparts. (D) Kaplan-Meier estimates of OS in the MSKCC non-ICI treatment cohort comparing patients with POL-MUT with their respective WT counterparts.

**Supplementary Figure 4. Predictive value of different POL-MUTs.** (A) Proportions of patients achieved objective response in POL-EDM, POL-nonEDM and POL-WT patients in TMB-high and TMB-low subgroup. (B) Kaplan-Meier estimates of PFS in the MSKCC cohort comparing POL-EDM, POL-nonEDM, and POL-WT patients in ICI treatment. (C) Kaplan-Meier estimates of OS in the MSKCC cohort comparing POL-EDM, POL-nonEDM, and POL-WT patients in cohort1.

**Supplementary Figure 5. Correlation among MSI, POL-EDM and POL-nonEDM.** (A) Venn plot denoting the overlap among MSI, POL-EDM and POL-nonEDM patients.

**Supplementary Table:**

**Supplementary Table 1. Information of collected ICI treatment cohorts.**

**Supplementary Table 2. POL-MUT in cBio-portal.**

**Supplementary Table 3. Frequency of POL-MUT in each cancer type.**

**Supplementary Table 4. TMB data using WES in cBio-portal.**

**Supplementary Table 5. TMB data using MSK-Impact in cBio-portal.**

**Supplementary Table 6. Lowest TMB detected in each POL-MUT.**

**Supplementary Table 7. Mutation Signatures in hypermutant POL-MUT patients with POL-PDS.**

**Supplementary Table 8: Specific POL-MUTs on patients deriving response or non-response from ICI treatment.**
