# Supplementary material for: POLE/POLD1 mutation in non‐exonuclease domain matters for predicting efficacy of immune‐checkpoint‐inhibitor therapy
Source: Clin Transl Med. 2021 Sep 26;11(9):e524. doi: 10.1002/ctm2.524 (PMC8473642; doi:10.1002/ctm2.524)

Supplementary Figure 1

A

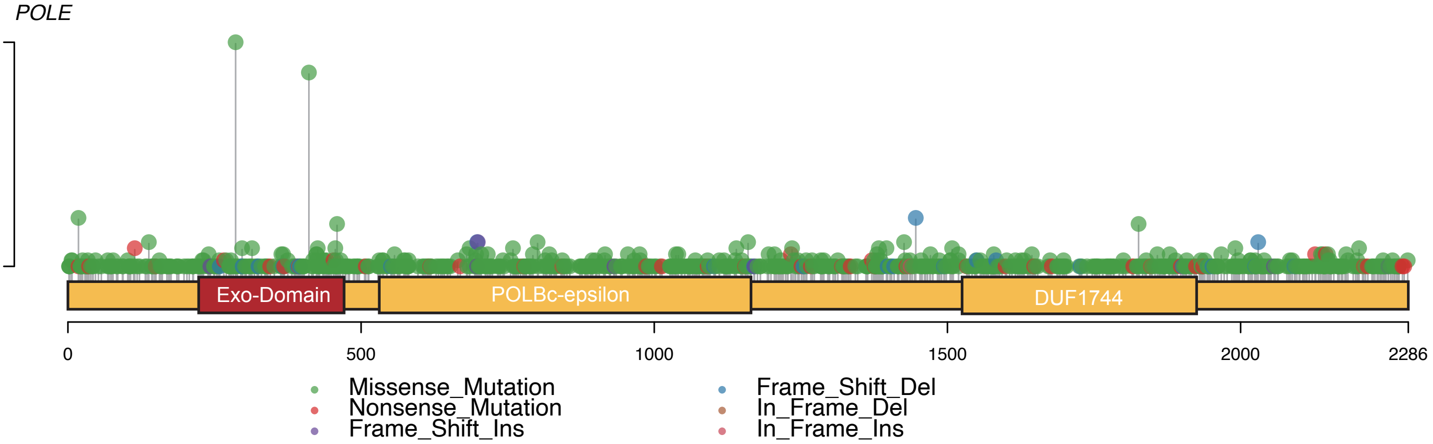

B

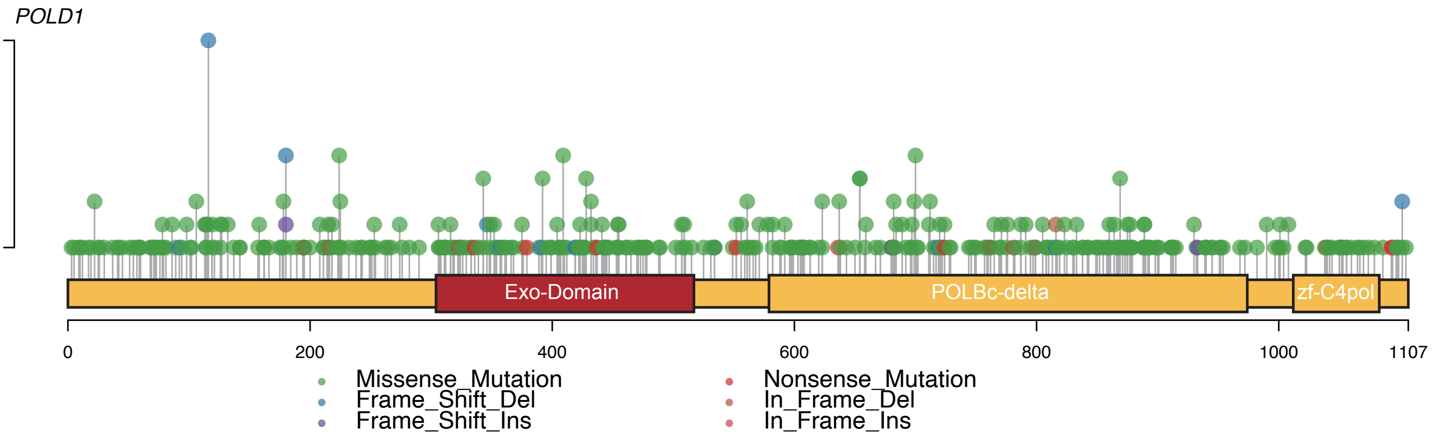

C

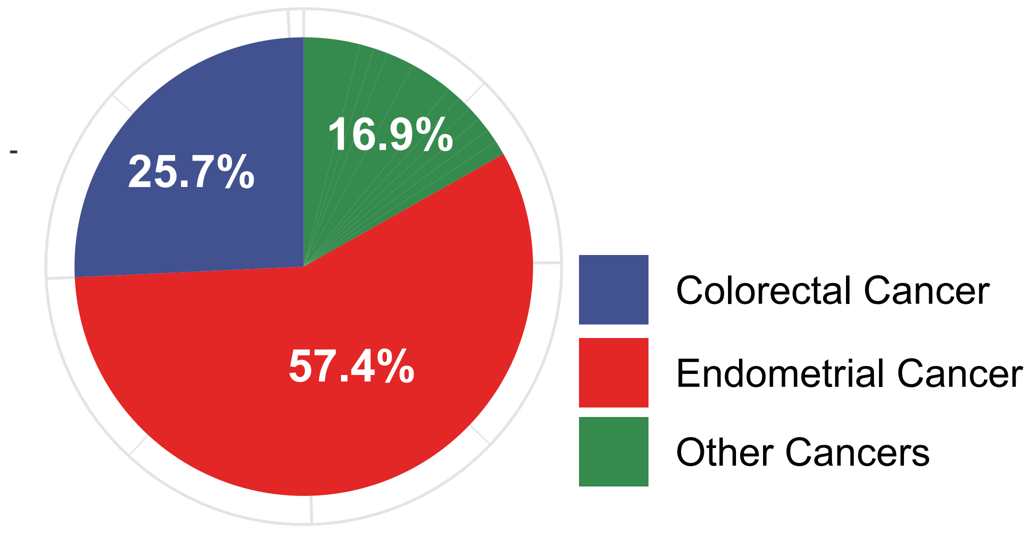

Supplementary Figure 2

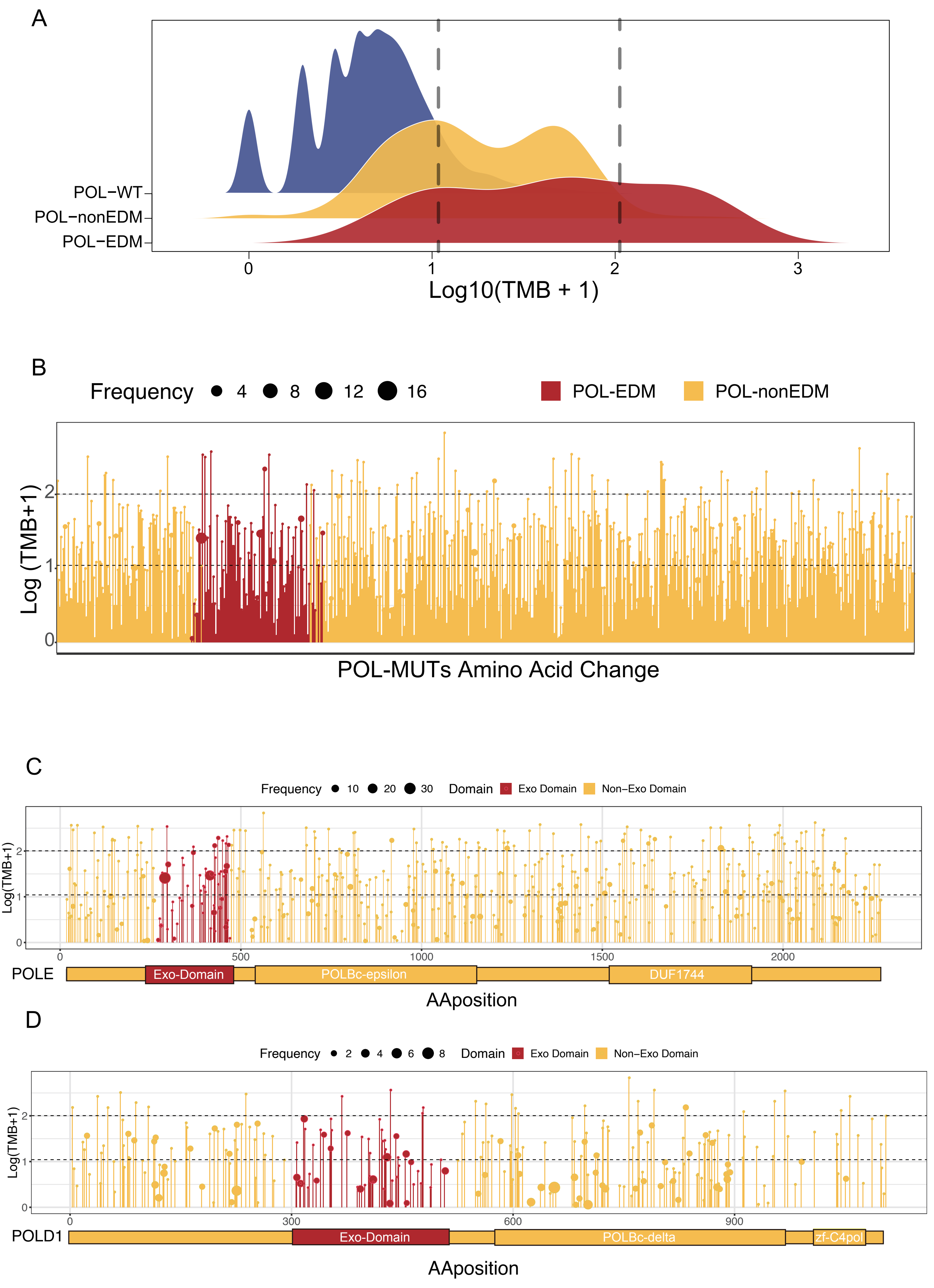

Supplementary Figure 3

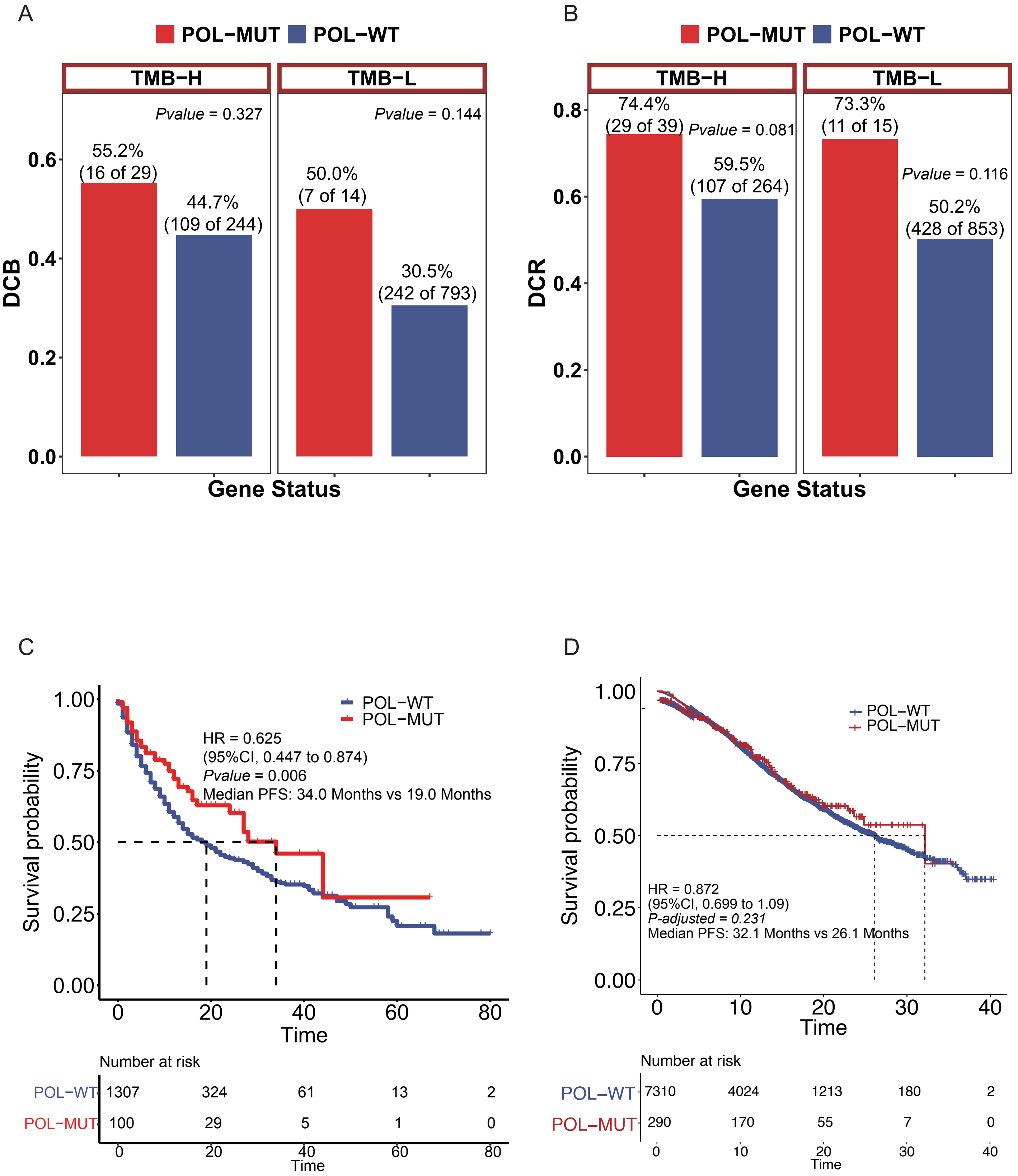

Supplementary Figure 4

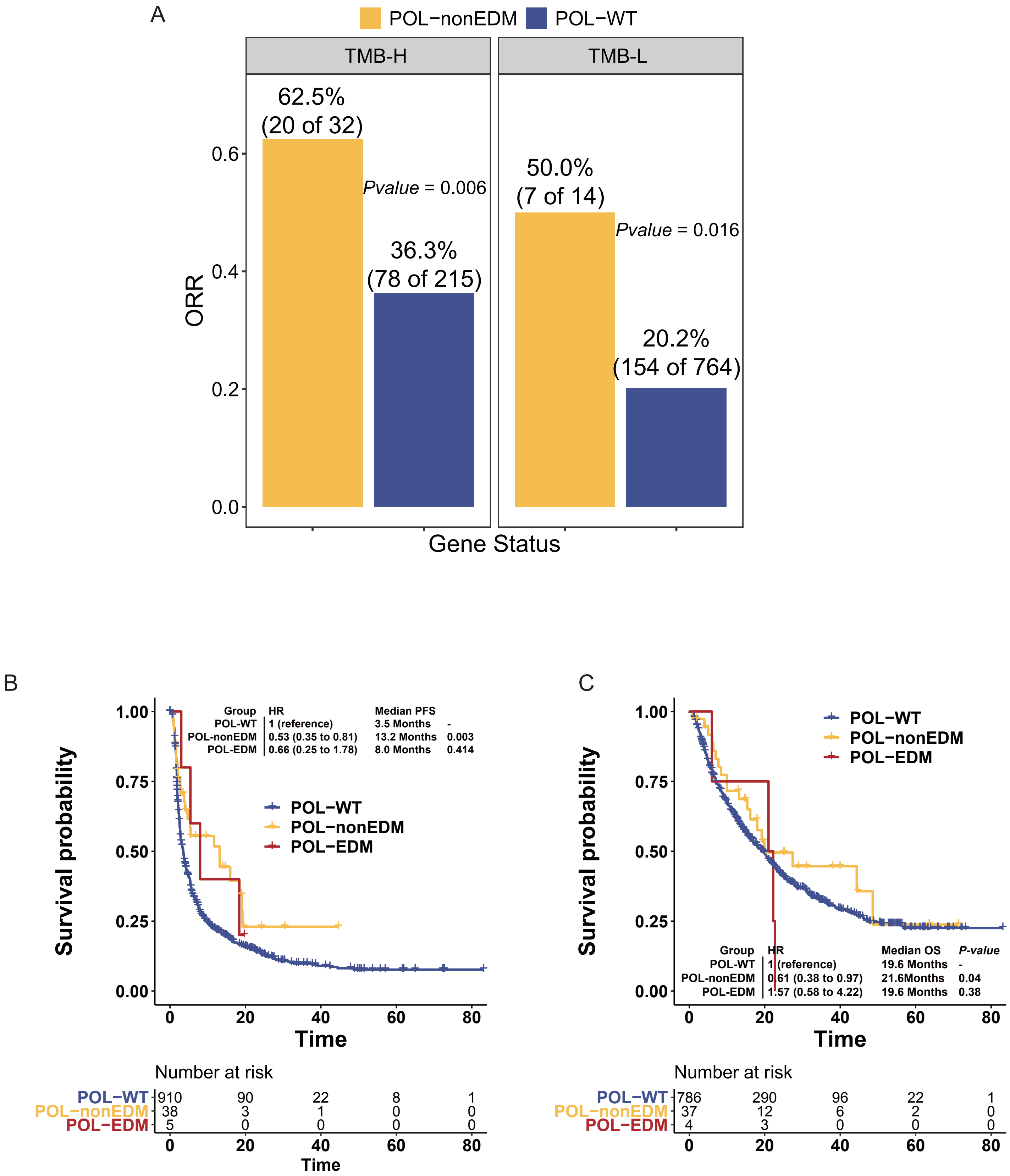

Supplementary Figure 5

A

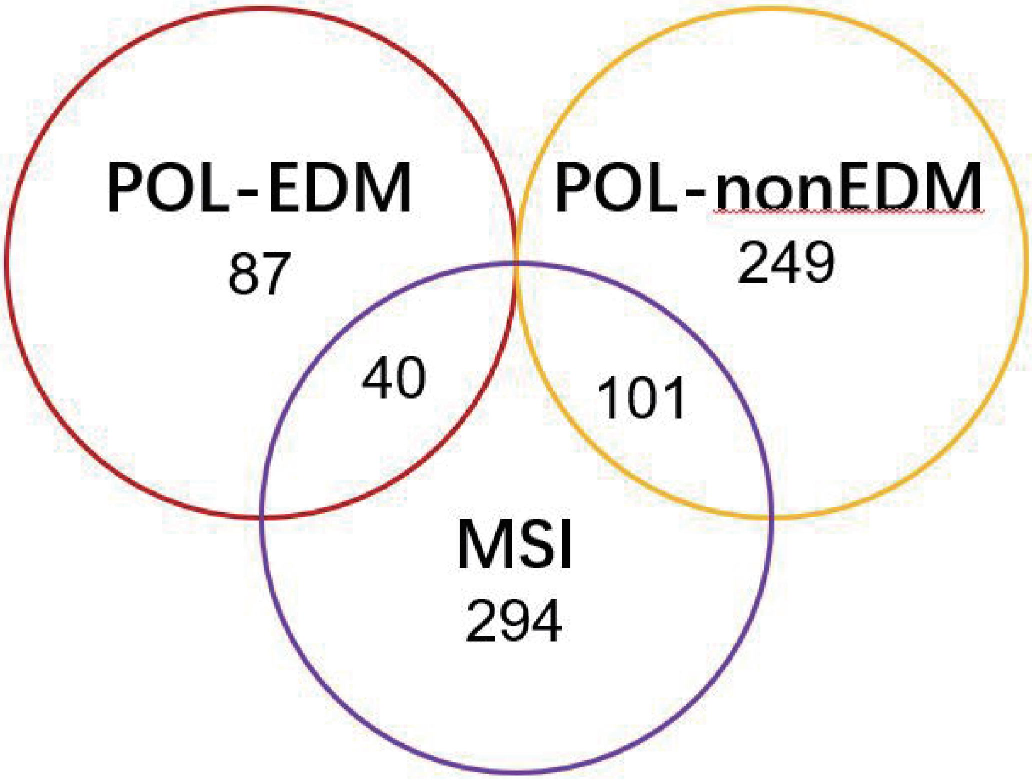

Supplement: Supplementary file 3 — Supplementary Figure [file CTM2-11-e524-s003.pdf]
